# Correlation of the self-reported Leeds assessment of neuropathic symptoms and signs score, clinical neurological examination and MR imaging in patients with lumbo-sacral radiculopathy

**DOI:** 10.1186/s12883-019-1333-3

**Published:** 2019-05-30

**Authors:** Nassib Tawa, Ina Diener, Quinette Louw, Anthea Rhoda

**Affiliations:** 1 Department of Rehabilitation Sciences, College of Health Sciences, Jomo Kenyatta University of Agriculture and Technology, PO Box 62 000 00200, Nairobi, Kenya; 2 Division of Physiotherapy, Faculty of Medicine and Health Sciences, Stellenbosch University, Private Bag X1, Matieland, 7602 South Africa; 3 Department of Physiotherapy, Faculty of Community and Health Sciences, University of the Western Cape, Private Bag X17, Bellville, 7535 South Africa

**Keywords:** Lumbar, Sacral, Radiculopathy, Diagnosis, Correlation

## Abstract

**Background:**

Lumbo-sacral radiculopathy (LSR) is a common musculoskeletal disorder for which patients seek medical care and referrals for advanced imaging. However, accurate diagnosis remains challenging. Neuropathic pain screening questionnaires, clinical neurological examination and magnetic resonance imaging (MRI) are used in the initial diagnosis. The utility of these tools in diagnosing LSR varies and their correlation has not been reported.

**Methods:**

A cross-sectional, multicentre, blinded design was used in six physiotherapy departments in Kenya. Each participant was blindly examined by three independent clinicians using the Self-Reported Leeds Assessment of Neuropathic Symptoms and Signs (S-LANSS) score, clinical neurological examination (CNE) and MRI. Spearman’s rank coefficient (*r)* was used to examine the correlation between the three tests. Linear regression and odds ratios were used to establish correlations between socio-demographic, clinical and diagnostic parameters. The diagnostic accuracy of individual or combined sets of CNE tests in diagnosing LSR, with reference to MRI, was determined using Receiver Operating Characteristics (ROC) curves.

**Results:**

We enrolled 102 participants (44 males, 58 females; mean age: 44.7 years). Results indicated a significant positive correlation (*r* = 0.36, *P* = 0.01) between S-LANSS, CNE and MRI among patients with low back and radiating leg symptoms. Positive agreement existed between combined neuro-conduction tests (sensory, motor and reflex) and neuro-dynamic tests (NDT).

The NDT component of CNE (Straight Leg Raise Test [SLRT] and Femoral Nerve Stretch Test [FNST]) was significantly associated (*P* = 0.05) with MRI: patients who had positive NDT results had higher odds (8.3) for positive nerve root compromise on MRI versus those who had negative NDT results.

**Conclusion:**

This was the first study to investigate the correlation between S-LANSS, CNE and MRI in patients presenting with low back and radiating leg symptoms. Results indicated a significant positive correlation. The strongest correlations to MRI findings of LSR were firstly, NDT (SLRT and FNST); secondly, the S-LANSS score; and thirdly, the CNE components of motor power and deep tendon reflex. The clinical implication is that clinicians can confidently use the S-LANSS score and CNE to diagnose and make therapeutic decisions in LSR, when MRI is medically contra-indicated, unaffordable or unavailable.

**Electronic supplementary material:**

The online version of this article (10.1186/s12883-019-1333-3) contains supplementary material, which is available to authorized users.

## Background

Lumbo-sacral radiculopathy (LSR) is a common condition encountered by clinicians in daily practice, but its diagnosis remains challenging [1]. Moreover, LSR impose a significant impact on patients’ health, socio-economic status, activity and participation levels and quality of life [1–4]. Common options available for diagnosing LSR include neuropathic pain screening questionnaires, clinical neurological tests and imaging [5, 6].

LSR has a unique pathophysiology, with clinical manifestation in characteristic patterns; indicating a specific underlying mechanism [7–9]. It therefore needs to be differentiated from somatic or visceral referred pain. Early and accurate diagnosis of LSR is important as current clinical practice highly advocates the differentiation of spinal pain according to the underlying mechanism and source.

Such differentiation informs therapeutic clinical decisions [3]. The effective management of acute lumbar spinal pain has been suggested as the best prevention for developing chronic pain [9].

In the initial diagnostic work-up of patients presenting with lumbo-sacral spinal pain, clinicians consider findings of various diagnostic procedures including neuropathic pain screening, clinical neurological examination, radiological imaging and electro-diagnostic studies [5, 6]. However, in the diagnosis of LSR, clinicians are encouraged to correlate findings of various diagnostic tools because of the shortcomings of individual tools and procedures [10–12].

The Self-Reported Leeds Assessment of Neuropathic Symptoms and Signs (S-LANSS) score is the most widely-used neuropathic pain screening questionnaire established in the literature and has significant levels of sensitivity and specificity in detecting LSR [13, 14]. Clinical neurological examination (CNE) is another method of diagnosing LSR. CNE is not only important for the identification of whether or not LSR is present, but also for anatomical localisation of radicular symptoms. If properly conducted, CNE could detect or exclude the presence of LSR based on characteristic physical findings [15, 16]. CNE has been reported to have a high prevalence rate for positive symptomatic findings and is often used for anatomical localisation of the symptomatic spinal structure responsible for patients’ radicular symptoms. This is important, especially when using targeted treatments like physiotherapy, manual therapy and surgery.

Magnetic resonance imaging (MRI), a relatively expensive and often unavailable option in resource-scare settings, has become a gold standard diagnostic measure among clinicians in diagnosing LSR [6, 17]. In fact, there is an emerging trend of over-utilisation and over-dependency on MRI in diagnosing LSR, which significantly impacts on the cost of care and, ultimately, patient outcomes [18].

Even though the accuracy of MRI in diagnosing disco-genic radicular symptoms has been reported [19, 20], it is known that LSR could also be caused by far-out extra-foraminal spinal stenosis lesions, which MRI cannot detect [21] and diagnostic inaccuracies have been reported in a recent study [12].

Therefore, the correct application and an understanding of the limitations of MRI examination is critical in the assessment of patients suspected with LSR [5, 6]. Similarly, in patients presenting with low back and radiating leg symptoms that are clinically consistent with LSR, but have negative MRI findings on lumbo-sacral nerve root compromise, other diagnostic measures for LSR should be considered. There is thus a need to know which diagnostic tools correlate with MRI in diagnosing LSR.

Diagnostic correlation between the three commonly-used procedures mentioned above (pain screening questionnaires, clinical examination and MRI) has not been empirically explored and documented. Therefore, this study aimed to determine the correlation between the S-LANSS, clinical neurological examination and lumbar MRI reports among patients presenting with low back and radiating leg symptoms at physiotherapy clinics in Kenya.

## Methods

### Design, setting and participants

We conducted a cross-sectional, multicentre, blinded study to examine the correlation of S-LANSS scores, CNE findings and MRI reports. Both test-execution and the interpretation of test results were conducted blindly. Data collection was separately and independently performed by pre-trained physiotherapists and radiologists in six different physiotherapy departments in the Republic of Kenya. This study was conducted using the Standards for Reporting Diagnostic Test Accuracy Studies (STARD) framework (Additional file 1).

We recruited both male and female patients aged 18 years and older. Participants had to present with an acute episode of low back and radiating leg symptoms below the gluteal fold, as diagnosed by the referring physician, at the time of data collection. We recruited patients who had been referred for physiotherapy treatment following an MRI examination done within the past 48 h. Patients were excluded if they had been diagnosed with a life-threatening comorbidity such as cancer, or serious medical and psychiatric conditions. Inability to read and write in English was also an exclusion criterion because of the need to complete the S-LANSS scale independently.

Sample size was informed by a retrospective review of physiotherapy admissions and attendance records of patients with low back and radiating leg symptoms at each of the six study centres for the preceding year (2013). The total average from all six study centres was then considered as the population (N) of patients with the target condition, from which the study sample (n) was derived using the Cochran formula [22]. This study therefore included 104 participants from the six centres. The study was jointly approved by the Senate Research Ethics Committee of University of the Western Cape and the Ethics Committee of the Kenya Medical Research Institute (Registration number 11/10/32).

### Procedure and protocols

**Fig. 1:**
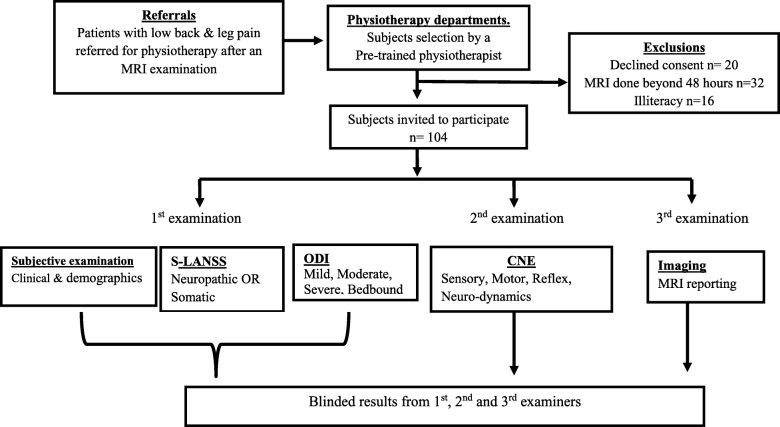
Data collection process

Data were collected from March to June 2014. For each participant, all three diagnostic assessment methods were performed within a period of 48 h. Data collection was conducted in three steps (Fig. 1). Firstly, a pre-trained physiotherapist, who was blind to the patients’ medical history and referring clinicians’ diagnosis, conducted a structured subjective examination (using a researcher-developed pain and socio-demographic questionnaire) and subsequently administered the S-LANSS and Oswestry Disability Index (ODI) questionnaire.

Secondly, a second pre-trained physiotherapist, who was blind to the patients’ subjective examination results, S-LANSS score and ODI report, conducted a structured CNE of the lumbo-sacral spine, using a standardised valid and reliable CNE protocol. Findings of this second examination on the same participant were documented in a CNE data sheet. Thirdly and finally, a radiologist interpreted the MRI film and completed the reporting according to a standard protocol. The radiologists who completed the MRI protocols were blind to the patient’s medical history and initial diagnosis.

### Statistical analysis

Data were analysed using SPSS version 21.0. Kolmogorov-Smirnov tests were performed to determine the distribution of the data and Spearman’s rank correlation coefficient *r* test was used to perform bivariate analysis. Linear regression and odds ratio analyses were performed to established possible correlations. Receiver Operating Characteristics (ROC) curves determined the agreement of individual or combined sets of CNE tests with MRI reports in detecting LSR.

## Results

Among a total sample of 102 participants, the mean age was 44.7 (range: 19–86) years. The sample had a gender distribution of 57% females and 43% males. Table 1 presents the socio-demographic and clinical characteristics and diagnostic findings of the study participants.

**Table 1:** Participants’ characteristics (*n* = 102)

Characteristic	Category	Percentage (%)
Gender, % females		57
Age, year (range)		44.7 (18–76)
Pain intensity (NPRS)	≤3 (mild pain)	5
	4–6 (moderate pain)	51
	7–9 (severe pain)	38
	=10 (excruciating pain)	6
S-LANSS score	Neuropathic	66
	Somatic	34
ODI score	Mild	13
	Moderate	36
	Severe	37
	Bed-bound	14
Neuro-conduction tests	Negative	32
	Mild deficit (one +ve test)	29
	Moderate deficit (two +ve tests)	18
	Severe deficit (three +ve tests)	21
Neuro-dynamic tests	Positive	63
	Negative	37
MRI-visible nerve root compromise	Negative	45
	Slight compromise	8
	Moderate compromise	19
	Severe nerve derangement	28

### Clinical findings

#### S-LANSS scores

Using a cut-off of 12 points for positivity of LSR by S-LANSS scale, results indicated that 66% (*n* = 102) of the participants were positive for LSR, while 34% (*n* = 102) were negative.

#### CNE tests

Sixty-eight percent (*n* = 102) of participants had positive nerve conduction tests results on the symptomatic side. Twenty-nine percent of participants had mild nerve conduction deficit (in this study defined as having a single positive test result). Moderate nerve conduction deficit (two positive test results during the clinical examination) was identified in 18%, while severe nerve conduction deficit (decrease in skin sensation, muscle power and deep tendon reflex tests) was present in 21%.

Results on neuro-dynamic testing of the lumbar and sacral nerve roots revealed positive neuro-mechano-sensitivity in a majority (64%, *n* = 102) of the sample, while 36% did not test positive for neuro-mechano-sensitivity.

#### MRI

Fifty-five percent (n = 102) of participants demonstrated positive MRI-visible nerve root compromise (defined as significant spinal canal stenosis and / or intervertebral disc (IVD) protrusion or protuberance). Forty-five percent had negative MRI findings on nerve root compromise, despite the classical presentations of lumbo-sacral spinal and referred leg symptoms.

### Bivariate analysis

#### Agreement between CNE and MRI findings on nerve root compromise

The agreement between nerve conduction and neuro-dynamic testing (categorical variables) and MRI findings on nerve root compromise (dichotomised into positive or negative) was assessed using the Spearman’s rank correlation coefficient *r.* Results indicated a positive relationship (*r* = 0.36, *P* = 0.01) between the two diagnostic tests (neuro-conduction and neuro-dynamic) and MRI. A positive agreement was also evident between combined neuro-conduction tests (sensory, motor and reflex) and neuro-dynamic testing (NDT; Straight Leg Raise Test [SLRT] and Femoral Nerve Stretch Test [FNST]). Individual CNE tests did not correlate well with MRI findings of nerve root compromise.

#### Logistic regression and odds ratio

Binary logistic regression analysis was used to explore the utility of S-LANSS scale and the various aspects of CNE in predicting the outcome of MRI reports on patients with clinical suspicion of LSR. Results indicated that the neuro-dynamic test component of the CNE, (in this study comprising the SLRT and FNST for lumbar and sacral nerve roots respectively) has a significant association (*P* = 0.05) with MRI findings. Patients who had positive neuro-dynamic test results were eight times more likely (odds ratio 8.3) to have positive reports of MRI-visible nerve root compromise compared to those who had negative neuro-dynamic test results.

No significant association was evident between CNE tests of nerve conduction (sensory, motor and reflex) in predicting the possible outcome of MRI in detecting nerve root compromise and radiculopathy. Table 2 presents the diagnostic predictive values and odds ratios.

**Table 2:** Diagnostic predictive values and odds ratios

Diagnostic test	Predictive value	*p*-value	Odds Ratio
S-LANSS score	−.534	.269	1.510
Sensory	−.260	.617	2.133
Motor	−.201	.692	2.210
Tendon reflex	−1.478	.010^a^	.698
Neuro-dynamics	1.155	.019^a^	8.301

^a^Statistically significant

### Agreement of S-LANSS and CNE with MRI

Table 3 presents the agreement of S-LANSS scores and CNE test findings with MRI reports in detecting LSR in measures of true positive (TP), false positive (FP), false negative (FN), and true negative (TN) alongside sensitivity, specificity, positive likelihood ratio (+ LR) and negative likelihood ratio (−LR).

**Table 3:** Diagnostic performance of S-LANSS and CNE compared to MRI

Diagnostic test	TP	FP	FN	TN	Sensitivity	Specificity	+LR	-LR
S-LANSS	42	25	14	21	0.75	0.6	1.87	2.4
Skin sensation	27	13	29	33	0.48	0.71	1.66	1.37
Motor power	35	16	21	30	0.63	0.65	1.8	1.76
Tendon reflex	29	6	27	40	0.52	0.87	4	1.8
LLNDTs	44	20	12	26	0.79	0.57	1.84	2.71

Key: *TP* True positive, *FP* False positive, *FN* False negative, *TN* True negative, *+LR* Positive likelihood ratio, *−LR* Negative likelihood ratio

Lower limb neuro-dynamic tests demonstrated the best sensitivity (0.79), followed by S-LANSS (0.75). Deep tendon reflex testing of the patellar and Achilles tendons were the most specific CNE (nerve conduction) tests (0.87).

## Discussion

This diagnostic test accuracy (DTA) study investigated the correlation of S-LANSS, CNE and MRI among patients presenting with low back and radiating leg symptoms consistent with LSR. Unlike most previous studies, this study defined lumbar nerve root compromise as significant spinal canal stenosis and / or disc protuberance, and not presence of disc prolapse on MRI. We used a cross-sectional, multicenter, blinded study design where subjects were separately examined by three independent pre-trained clinicians who were blind to the results of other examinations.

To our knowledge, this was the first study to investigate correlation of three commonly-used diagnostic tools employed in the assessment of lumbar spinal pain patients.

We found a significant positive correlation between S-LANSS, CNE and lumbar MRI findings. The strongest correlation to MRI findings were firstly, NDT (SLRT and FNST); secondly, the S-LANSS score; and thirdly, the CNE components of motor power and deep tendon reflex. There was also a positive agreement between combined neuro-conduction tests (sensory, motor and reflex) and NDT (FNST and SLRT).

Clinically, these observations imply that S-LANSS scale and valid, reliable CNE tests could be used to diagnose and make therapeutic decisions on LSR in the event that MRI is medically contra-indicated, unaffordable or even unavailable.

Our findings support previous reports [13, 22] regarding the use of quick, low-risk and cost-effective diagnostic options in the assessment of lumbar spinal pain patients, especially in primary care settings of resource-poor countries of sub-Sahara Africa. Furthermore, because MRI is not recommended within the first 4 to 6 weeks of an acute episode of low back pain [4, 5], S-LANSS and CNE tests may be used during this period to confirm or refute a clinical suspicion of LSR.

Our findings agree with those by Bertilson et al. [17] regarding correlation between MRI and pain drawing, given that one section of the S-LANSS scale and pain drawing both involve mapping of the area(s) of pain or discomfort on a body chart and use of pain descriptors like “numbness” and “stinging”. However, a previous correlation study [23] reported that MRI findings of nerve root involvement showed no significant correlation with CNE tests of muscle weakness. This discrepancy may stem from the fact that in the previous study [23], CNE was done with prior knowledge of the MRI findings: a possible source of verification bias, which has been reported by another similar study [24]. In addition, comparing MRI to a single motor test, as in [23], does not necessarily reflect the actual clinical practice.

Our findings are clinically relevant as we established a significant positive correlation between S-LANSS, CNE and MRI, which may inform clinicians’ decisions regarding the diagnosis and management of LSR. We furthermore established the most sensitive test combinations of CNE as being neuro-dynamic, motor and deep tendon reflex tests.

A potential limitation of this study is that most of the physiotherapists conducting the structured CNE only learnt to do so specifically for this study, and were thus not well-experienced. Despite pre-training, this may have negatively impacted on the quality of test-execution and interpretation of findings. Future studies should investigate the correlation of these tools in diagnosing level-specific lumbar or cervical nerve root compromise and radiculopathy, using examiners who have specialised training and experience in spinal musculoskeletal health.

## Conclusion

The S-LANSS and lumbar CNE tests correlate positively to MRI findings in diagnosing LSR among patients with low back and radiating leg pain. NDTs (SLRT and FNST) demonstrate the strongest correlation to MRI, followed by S-LANSS score and motor power and deep tendon reflex tests. Our findings suggest that clinicians could diagnose and make therapeutic decisions for patients presenting with low back and referred leg pain based on the findings of these rapid, cost-effective and user-friendly tests, should confirmatory MRI be medically contra-indicated, unaffordable or unavailable. This finding is especially valuable in the context of resource-poor primary care settings of low-income countries like Kenya.

## Additional file

Additional file 1: Standards for Reporting Diagnostic Test Accuracy Studies (STARD) framework. The additional file one describes the application of the STARD framework guidelines in the study. (DOCX 14 kb)

## Abbreviations

CNE: Clinical neurological examination
DRG: Dorsal root ganglion
DTA: Diagnostic test accuracy
FNST: Femoral nerve stretch tests
IVD: Inter-vertebral disc
LSR: Lumbo-sacral radiculopathy
MRI: Magnetic resonance imaging
NDTs: Neuro-dynamic Tests
ODI: Oswestry Disability Index
S-LANSS: Self-reported Leeds Assessment of Neuropathic Symptoms and Signs Score
SLR: Straight Leg Raise Test
STARD: Standards for Reporting Diagnostic test accuracy Studies framework
