# Supplementary material for: Correlation of the self-reported Leeds assessment of neuropathic symptoms and signs score, clinical neurological examination and MR imaging in patients with lumbo-sacral radiculopathy
Source: BMC Neurol. 2019 May 30;19:107. doi: 10.1186/s12883-019-1333-3 (PMC6542141; doi:10.1186/s12883-019-1333-3)
Supplement: Supplementary file 1 — Standards for Reporting Diagnostic Test Accuracy Studies (STARD) framework. The additional file one describes the application of the STARD framework guidelines in the study. (DOCX 14 kb) [file 12883_2019_1333_MOESM1_ESM.docx]

**Additional file 1: Application of the STARD framework guidelines**

| STARD CRITERION | STARD GUIDELINES | APPLICATION IN THE STUDY |
| --- | --- | --- |
| 1 | Specification of clinical examiner’s attributes in terms of profession, qualifications and clinical experience. | Clinical examiners included Physiotherapists and radiologists who had clinical experience of 5 years and above. The physiotherapists had a minimum of a college diploma while the radiologists Masters in diagnostic radiology. |
| 2 | Clear description of patient’s attributes including age and gender | Inclusion criteria were set for both male and female patients aged 18 years and older. |
| 3 | Clarity and specification of the target condition | Only subjects with low back pain and referred leg symptoms, which is a clinical suspicion of LSR were included in the main study. |
| 4 | Standardisation of measurement tools and processes through pre-training of participating clinicians | The participating physiotherapists were pre-trained on the lumbar CNE protocol and inter-examiner reliability was established prior to the main study. The participating radiologists were also pre-trained on the lumbar MRI reporting protocol, and inter-rater reliability was established prior to the main study. |
| 5 | Acceptable time-lapse between the index test and the comparator test or reference standard | The S-LANSS and CNE which comprised the index tests were performed on the same day, while the MRI (reference standard) had been performed maximally 48 hours prior. |
| 6 | Blinding of clinical examiners | The 1^st^ examination was the S-LANSS which was patient self-administered and the results were collected by an independent research assistant. The 2^nd^ examination was CNE which was performed by an independent physiotherapist blind to the S-LANSS score. The 3^rd^ examination was lumbar MRI reporting by an independent pre-trained radiologist blind to both the S-LANSS and CNE results. The principal researcher separately received the three sets of results from the collection points. |
